# Genetic Risk Assessment of Nonsyndromic Cleft Lip with or without Cleft Palate by Linking Genetic Networks and Deep Learning Models

**DOI:** 10.3390/ijms24054557

**Published:** 2023-02-25

**Authors:** Geon Kang, Seung-Hak Baek, Young Ho Kim, Dong-Hyun Kim, Ji Wan Park

**Affiliations:** 1 Department of Medical Genetics, College of Medicine, Hallym University, Chuncheon 24252, Republic of Korea; 2 Department of Orthodontics, School of Dentistry, Seoul National University, Seoul 03080, Republic of Korea; 3 Department of Orthodontics, The Institute of Oral Health Science, Samsung Medical Center, School of Medicine, Sungkyunkwan University, Seoul 06351, Republic of Korea; 4 Department of Social and Preventive Medicine, College of Medicine, Hallym University, Chuncheon 24252, Republic of Korea

**Keywords:** artificial neural network, genetic algorithm, genetic risk prediction, neural networks ensemble, machine learning, nonsyndromic cleft lip with or without cleft palate, polygenic risk score, single nucleotide polymorphism

## Abstract

Recent deep learning algorithms have further improved risk classification capabilities. However, an appropriate feature selection method is required to overcome dimensionality issues in population-based genetic studies. In this Korean case–control study of nonsyndromic cleft lip with or without cleft palate (NSCL/P), we compared the predictive performance of models that were developed by using the genetic-algorithm-optimized neural networks ensemble (GANNE) technique with those models that were generated by eight conventional risk classification methods, including polygenic risk score (PRS), random forest (RF), support vector machine (SVM), extreme gradient boosting (XGBoost), and deep-learning-based artificial neural network (ANN). GANNE, which is capable of automatic input SNP selection, exhibited the highest predictive power, especially in the 10-SNP model (AUC of 88.2%), thus improving the AUC by 23% and 17% compared to PRS and ANN, respectively. Genes mapped with input SNPs that were selected by using a genetic algorithm (GA) were functionally validated for risks of developing NSCL/P in gene ontology and protein–protein interaction (PPI) network analyses. The *IRF6* gene, which is most frequently selected via GA, was also a major hub gene in the PPI network. Genes such as *RUNX2*, *MTHFR*, *PVRL1*, *TGFB3*, and *TBX22* significantly contributed to predicting NSCL/P risk. GANNE is an efficient disease risk classification method using a minimum optimal set of SNPs; however, further validation studies are needed to ensure the clinical utility of the model for predicting NSCL/P risk.

## 1. Introduction

Orofacial clefts (OC), which are the second most common congenital anomaly with a wide range of etiologies, can occur as an isolated form or as a syndrome. The prevalence of OC varies by region and ethnicity, with the highest incidence being observed in Asian populations [1]. According to a nationwide cohort study, the overall prevalence of OC in Korea was 1.96 per 1000 live births, and approximately 76.45% of all cases occur in the nonsyndromic form. Specifically, cleft lip only (CL), cleft lip with cleft palate (CLP), and cleft palate only (CP) accounted for 26.47%, 26.56%, and 52.97% of total cases, respectively [2]. As CP has been considered a distinct malformation, recent genetic studies have primarily focused on nonsyndromic cleft lip with or without cleft palate (NSCL/P), which is known to be more heritable [3].

From the 1990s to the early 2000s, family-based studies have provided evidence that chromosomal regions (such as 2p, 4q, and 6p) and genes (such as *COL11A1* and *TGFA*) are linked with nonsyndromic OCs. However, genetic association studies have shown much greater statistical power in detecting susceptibility genes for complex diseases, and genes involved in craniofacial development, such as *IRF6* and *MSX1*, have been identified to be associated with NSCL/P [4]. Since around 2010, genome-wide association studies (GWASs), which represent a hypothesis-free approach using millions of single nucleotide polymorphism (SNP) markers, have identified novel loci for NSCL/P, such as 8q24, 10q25.3, and 17q22 [5,6,7]. Although previous studies have been primarily conducted in populations with European ancestry, genetic heterogeneity among ethnic groups has become a major concern in identifying susceptibility variants for NSCL/P, as reported in a study of 8q24.21 and a Chinese GWAS [8,9].

With the accumulation of susceptibility SNPs discovered in GWAS, the demand for developing methods for predicting genetic risk is rapidly growing. Polygenic risk scoring (PRS), which is defined as a weighted sum of individual risk alleles, has been widely applied to predict multifactorial disease risk; however, its reliance on an additive model limits its application to elucidate complex interactions among genetic variants [10,11]. Furthermore, machine learning (ML) algorithms have been applied for the risk prediction of complex diseases, due to their strength in identifying patterns and interactions among multiple inputs by employing multivariate, nonparametric methods [12]. Zhang et al. (2018) evaluated seven ML techniques, including random forest (RF) and artificial neural network (ANN), by using forty-three NSCL/P-associated SNPs and reported that the logistic regression model had the highest classification performance in Han Chinese (AUC of 0.90) [13]. In a Brazilian study, RF and ANN effectively classified NSCL/P patients and normal subjects with greater than 94% accuracy by using 13 SNPs [14].

The recent advent of deep learning (DL) has further improved the classification capability for a disease by using individual SNP data, as was observed in a case–control study on obesity (AUC of 0.99) [15,16]. DL has been shown to be superior in mapping complex non-linear interactions and for integrating different types of data [17,18]; however, highly complex networks demand a large dataset to ensure sufficient predictive power and generalization of results [19,20,21]. Especially, given the difficulty of obtaining large numbers of human samples in the field of genomic medicine, appropriate feature selection directly affects model performance by reducing the noise and dimensionality of data in both traditional ML- and DL-based risk prediction methods [22,23,24]. The genetic algorithm (GA) is a promising method for optimizing feature selection. Tong and Schierz (2011) have successfully applied a hybrid genetic algorithm neural network (GANN) to extract highly informative genes from a microarray-based gene expression dataset [25]. In a separate study, Zhang et al. (2015) improved the performance of predicting immunogenic T-cell epitopes from epitope sequences through the use of an ensemble RF model that was trained on individual features selected with GA [26].

To the best of our knowledge, this study represents the first application of the GANNE approach to disease risk assessment and the first genetic risk prediction study for NSCL/P in the Korean population. Herein, we first performed a genetic association analysis by using 92 SNPs that were genotyped in 143 Korean children with NSCL/P and 119 healthy controls. We subsequently compared the predictive performance of the PRS and various ML methods. To improve predictive power, we proposed the use of a deep learning model that uses automatic feature selection for NSCL/P classification; specifically, we used the genetic-algorithm-optimized neural networks ensemble (GANNE). Finally, we functionally validated the genes selected by GANNE using pathway and network analyses.

## 2. Results

### 2.1. Genetic Association Analysis for NSCL/P

Four SNPs (rs10790330, rs906830, rs17104928, and rs3917211) demonstrated HWE *p*-values less than 0.05; however, none of the SNPs showed evidence of deviation from HWE (*p* > 0.01) in the control data, and the MAFs of all ninety-two SNPs were >1% in both the case and control groups. In the Fisher’s exact test, two intronic SNPs of *IRF6* in linkage disequilibrium (LD) with a *r*^2^ value of 0.80 (rs2235373 and rs2235371) were found to be significantly associated with NSCL/P (*p* = 3.5 × $10^{-4}$ and *p* = 4.5 × $10^{-4}$, respectively). Moreover, SNPs located near or within five other genes (*RUNX2*, *ARNT*, *TGFB3*, *MTHFR*, and *TCOF1*) also showed significant associations in Korean NSCL/P patients (*p* < 0.05) (Table 1). After accounting for pairwise LD ($r^{2}$ < 0.8, see Table S1), we identified three SNPs that were associated with NSCL/P at the level of *p* < 0.01, as well as ten SNPs with nominal significance (*p* < 0.05) and sixteen SNPs with marginal significance (*p* < 0.1).

### 2.2. Genetic Risk Prediction

**Figure 1:**
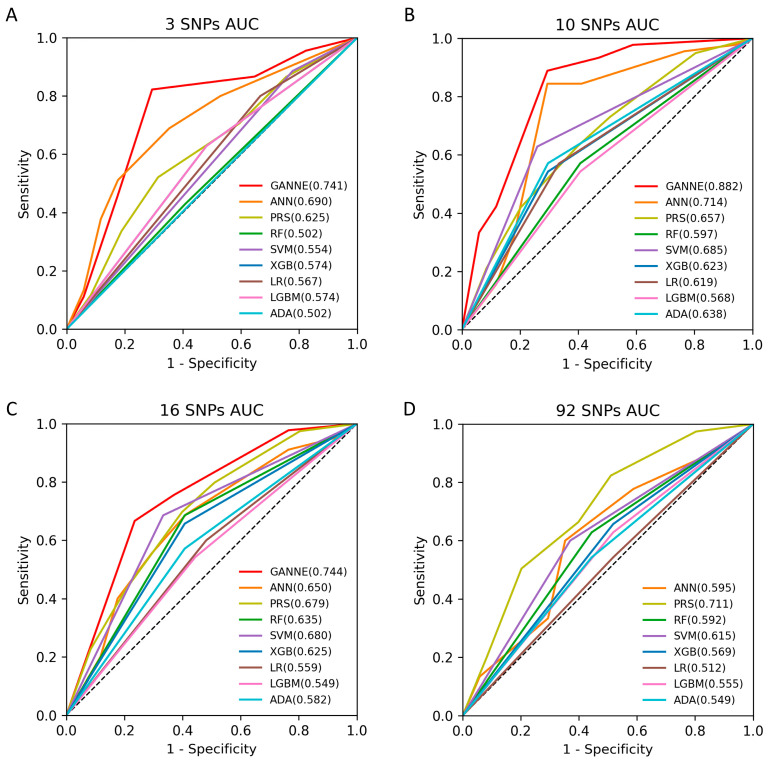
Comparison of predictive power by using nine models for nonsyndromic cleft lip with or without cleft palate risk. Model performance was measured by the area under the receiver operating curve (AUC) for each risk model in four different sets of single nucleotide polymorphisms (SNPs). (**A**) models trained on 3 SNPs with *p* < 0.01, (**B**) models trained on 10 SNPs with *p* < 0.05, (**C**) models trained on 16 SNPs with *p* < 0.1, and (**D**) models trained on all 92 SNPs. ADA, adaptive boosting; GANNE, genetic-algorithm-optimized neural networks ensemble; ANN, artificial neural network; PRS, polygenic risk score; RF, random forest; SVM; support vector machine; XGB, extreme gradient boosting; LR, logistic regression; LGBM, light gradient boosting model.

The predictive performance of the PRS models for NSCL/P risk increased as the number of SNPs increased (accuracy = 0.676 and AUC = 0.711 for the 92-SNP model). When evaluating the models generated by the six traditional machine learning algorithms, the training accuracies significantly improved to above 95% for the 10-SNP model, especially for four of the ML algorithms. However, the testing accuracies remained in the 60% range. Out of the 18 models categorized by the number of SNPs and the type of machine learning algorithm, the SVM utilizing 10 SNPs demonstrated the highest predictive performance (test accuracy = 0.677, F1 = 0.678, AUC = 0.685). On the other hand, LightGBM demonstrated the lowest predictive performance among the machine learning algorithms (test accuracy = 0.565, F1 = 0.566, AUC = 0.568). We trained the four sets of SNPs by using the ANN deep learning algorithm but did not observe a significant improvement in predictive performance compared to PRS and the machine learning models (test accuracy = 0.63, F1 = 0.65, AUC = 0.71) (Figure 1).

In the current study, we developed a model to improve NSCL/P classification by using the GANNE algorithm. We first prepared a set consisting of the top SNPs that were identified in the genetic association analysis, along with five optimal sets of SNPs that were selected by using GA, to be used as inputs for ANN deep learning. GANNE significantly improved predictive performance across all three SNP settings, especially the best model selected from six sets of ten SNPs (AUC of 88.2%), which increased AUC (∆AUC) by 17%, 23%, and 28.5%, respectively, compared to ANN, PRS, and RF (Figure 1). Despite the lower weighted F1-score of 0.76 compared to AUC, the 10-SNP GANNE model still demonstrated superior performance when accounting for class imbalance in the binary data. In addition, the test accuracy of the 10-SNP GANNE (74.2%) increased within the range of 6.5% (SVM) to 14.5% (RF) compared to other methods, and it increased by 11.3% compared to the deep-learning-based ANNs. GANNE models with three SNPs and sixteen SNPs exhibited similar test results (accuracy = 0.694, F1 = 0.709, AUC of approximately 0.744), but the 16-SNP GANNE demonstrated better training accuracy than the 3-SNP GANNE (Figure 1, Table 2).

The GANNE utilized 46, 25, and 15 different SNPs that were located in 14, 12, and 8 genes, respectively, at least once for the 3-, 10-, and 16-SNP models. Five SNPs from *IRF6* (including rs2013162), rs11204737 (*ARNT*), rs7715100 (*TCOF1)*, rs16873348 (*RUNX2*), and rs3917192 (*TGFB3)* were used in all three SNP models. Among the SNPs that were selected for the 10-SNP GANNE models, rs2013162 (*IRF6*) was the most potent SNP included in all six sets, followed by rs3917192 (*TGFB3*) in five sets (Table S2).

To verify the reproducibility of the deep learning models, we performed 100 iterations, and the average of the results in each iteration followed the trend of the best model results for each set of SNPs. As expected, the 10-SNP GANNE model produced the highest accuracy and AUC, even at 100 iterations (average training accuracy = 92.1%, average test accuracy = 65.4%, average test AUC = 75.2%), with the highest AUC of 89.5% (Table 2).

### 2.3. In Silico Functional Analysis

By using DAVID, we identified a total of 52 GO terms that were significantly associated with 12 genes harboring 25 SNPs used at least once in the 10-SNP GANNE (*p* < 0.05 and FDR < 0.1). In particular, the most enriched GO term (GO:0009888~tissue development) was associated with the following nine genes: *IRF6*, *RUNX2*, *TBX22*, *MTHFR*, *PVRL1*, *PAX9*, *TGFB3*, *TCOF1*, and *VAX1.* In addition, four genes (*RUNX2*, *PVRL1*, *PAX9*, and *TGFB3*) showed significant enrichment in GO:0042476~odontogenesis.

**Figure 2:**
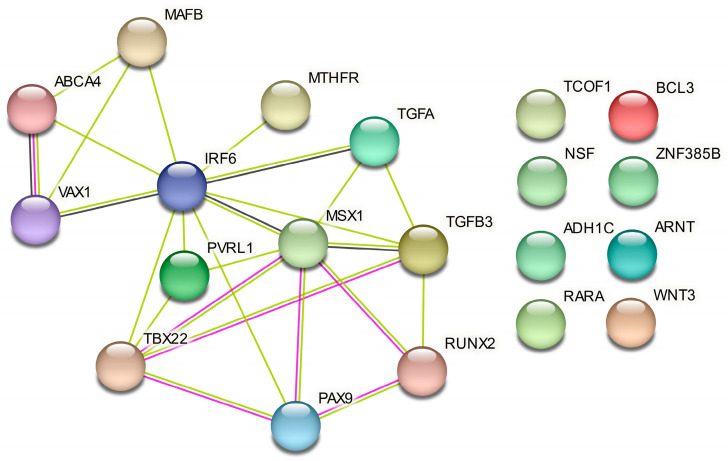
Protein–protein interaction network of 20 candidate genes for NSCL/P. Green line, the presence of co-publications in text mining; purple line, experimental evidence of co-expression; black line, evidence of mRNA co-expression (based on a STRING confidence score of 0.45).

In the PPI network analysis, nine of the twenty candidate genes that were evaluated in this study showed multiple interactions with other genes based on experimental evidence of co-expression. In particular, *MSX1* and *IRF6* were the most important hubs in this network, and genes such as *PAX9*, *TBX22*, *RUNX2*, *TGFB3*, and *VAX1* also appeared to interact with more than one gene. However, eight genes (*TCOF1*, *NSF*, *ADH1C*, *RARA*, *WNT3*, *ARNT*, *ZNF385B*, and *BCL3*) did not show an interaction at a confidence score of 0.45 (Figure 2).

## 3. Discussion

As the discovery of genetic variants associated with complex diseases increases, the demand for personalized health care services using genetic information is also rapidly increasing. To overcome the limitations of regression-based PRS and conventional ML algorithms, artificial intelligence (AI) has recently begun to be applied to risk prediction and the early diagnosis of complex diseases [11]. Unlike traditional machine learning algorithms, deep learning is helpful in solving complex problems with far more parameters but requires a large-scale dataset to avoid overfitting and to generalize results [27]. Therefore, state-of-the-art deep learning algorithms are not widely applied in genomic medicine due to the difficulty of large-scale sample collection.

In the current study, we improved the classification ability for NSCL/P in Korean individuals by performing a deep-learning-based ANN with informative SNPs selected via GA to reduce dimensionality while also increasing test accuracy. GANNE performed best for all three SNP settings compared to the eight conventional methods for risk prediction. In conjunction with the results of the in silico functional analysis, we also demonstrated the possibility of explaining interactions among genetic features, which have been considered a black box in ML applications.

The machine learning algorithms, including GANNE, showed the highest classification accuracy when using 10 SNPs but the performance declined as the number of input SNPs increased. On the other hand, PRS, a widely used method in predicting complex disease risk, exhibited a consistent improvement in its AUC with the addition of more SNPs. Despite the simplicity in implementation, logistic-regression-based methods, such as LR and PRS, may not be effective in dealing with non-linear or highly correlated input data [10]. Our findings underscore the issue of dimensionality, whereby the number of required datasets increases exponentially as the input dimensionality increases when using ML algorithms as genetic risk predictors [24]. Supervised machine learning algorithms, RF and SVM, tend to perform well in high-dimensional data, but are prone to overfitting and are computationally intensive [12]. In this study, we found SVM to be more suitable for the non-linear binary classification task, as it showed better predictive performance (F1 = 0.678) compared to RF (F1 = 0.598). Boosting algorithms, including XGBoost, Adaboost, and LightGBM, are ensemble techniques that combine multiple models with weak predictive performance to form a more potent model [28]. Among the nine classification methods used in this study, LightGBM exhibited the lowest predictive performance. Further studies are necessary to investigate the impact of the strengths and limitations of each ML algorithm on disease risk prediction accuracy.

There have been attempts to improve predictive accuracy by combining results from different SNP models, but most statistical association analyses have limitations in selecting different subsets of SNPs [29]. Although there are 7 trillion possibilities to select a set of 10 SNPs out of 92 SNPs in our dataset, GANNE can efficiently select an optimal set of SNPs by initializing the first population with the best SNPs that were identified in the association analysis.

In particular, the 10-SNP GANNE model showed excellent performance and improved the AUC by 28.6%, 23%, and 17% compared to the RF, PRS, and ANN methods, respectively, by including SNPs that did not show a strong association with NSCL/P, which was likely due to a lack of statistical power. GA selected the SNPs that were significantly associated with NSCL/P while also extracting SNPs (such as rs7103685 in the *PVRL1* gene) that did not show significant associations but that were used in four of the six SNP sets (*p* = 0.46).

Although a further evaluation of gene–gene interactions by using PLINK did not yield statistical significance, a functional protein association network analysis suggests that GA considers functional interactions of genes in SNP selection. The *IRF6* gene that was most frequently selected by GA was also a major hub gene in the PPI network, and its association with NSCL/P has been reported in previous studies [30]. However, *MSX1*, which is another hub gene in the PPI network, was selected by GA in the 16-SNP subset but not in the 10-SNP subset. Moreover, all three SNP markers for the *MSX1* gene were not statistically significant in this case–control analysis, but its association with NSCL/P remains controversial with inconsistent results, especially in Asian studies [31,32,33]. GANNE has demonstrated the potential to identify significant interactions among genes when used in conjunction with the PPI network analysis. Due to the fact that there may be valid interactions between SNPs that cannot be detected by using statistical analysis, neural-network-based genetic interaction studies using tools such as class activation mapping or attention modules may be needed in the future [34].

In this study, we demonstrated that GANNE, which is an ensemble neural network with automated feature selection, outperforms existing methods in predicting NSCL/P risk with genotype data by reducing the input dimension of each network through the use of a GA. Although GANNE achieves better generalization and robustness than other classification methods, given the number of samples that were trained in this study, further studies with larger samples are needed to validate the accuracy of the model. In genetic association studies, adjustments for age as a potential confounder are usually unnecessary, as differences in age between cases and controls may be associated with disease outcome but unlikely with genotype [35].

## 4. Materials and Methods

### 4.1. Study Subjects

We evaluated 143 Korean NSCL/P patients (91 males and 52 females) from 258 Korean families with nonsyndromic OC who visited Seoul National University Dental Hospital and SAMSUNG Medical Center. At each hospital, an orthodontist diagnosed the types of NSCL/P in the cases (nine cases with cleft lip only, twenty-six cases with cleft lip and alveolus, and one hundred and eight cases with cleft lip and palate). As a control group, we selected 119 healthy Korean adults without OC (60 males and 59 females) from a community-based cohort that was jointly developed by Hallym University College of Medicine and Chuncheon Sacred Heart Hospital. A trained dentist or clinician interviewed the participants and collected peripheral venous blood samples after obtaining informed written consent. The Institutional Review Board of each institution approved this study protocol. The details of the data collection can be found elsewhere [36,37].

### 4.2. SNP Genotyping

By using literature reviews, we identified nineteen candidate genes, including *PAX9* and *TGFA*, and two chromosomal loci (8q24.21 and 10q250), which have been reported to be associated with NSCL/P in previous studies. By using a web browser known as, ‘TAG SNP selection (TagSNP)’ (https://snpinfo.niehs.nih.gov/snpinfo/snptag.html) [38], we identified SNP markers that were frequently found in East Asian populations among SNPs located within 2 kb from each of the 5′ and 3′ ends of the candidate genes. Genomic DNA was isolated from each blood sample by using a commercial DNA extraction kit (Quiagen Inc., Valencia, CA, USA) at the Samsung Biomedical Research Center, and genotype data were generated via SNP Genetics Inc. (Seoul, Republic of Korea) by using VeraCode Technology (Illumina Inc., San Diego, CA, USA). Details of these procedures are presented elsewhere [39].

### 4.3. Genetic Association Analysis

We subsequently analyzed only 92 SNPs in Hardy–Weinberg equilibrium (HWE *p*-values greater than 0.01) with both genotype and sample call rates greater than 95% and a minor allele frequency (MAF) greater than 1%. After SNP quality control, a pairwise LD was estimated by calculating *r^2^* via the Haploview program in the control group. The missing genotypes were imputed by considering the calculated LD [40]. We performed Fisher’s exact test by using PLINK 1.9 for genetic association analysis [41].

### 4.4. Genetic Risk Prediction

#### 4.4.1. SNP Subset Selection

Based on the statistical significance obtained by the Fisher’s exact test, we selected four subsets of SNP markers for the binary classification of NSCL/P risk: three SNPs (*p* < 0.01), ten SNPs (*p* < 0.05), sixteen SNPs (*p* < 0.1), and ninety-two SNPs (all). SNPs in LD (*r*^2^ > 0.8) were excluded (except for the 92-SNP set). Of the 262 samples, we used 200 samples (100 cases and 100 controls) in the training process (180 samples for training and 20 samples for validation) and 62 samples (43 cases and 19 controls) for testing purposes.

#### 4.4.2. Polygenic Risk Score

We calculated the PRS for each *j*th subject by using the equation $PRS_{j}=\overset{M}{\underset{i=1}{\sum }}(logOR_{i}\times x_{ij})$, where M is the number of SNP markers, $logOR_{i}$ is the natural logarithmically transformed odds ratio (OR) of the *i*th susceptibility SNP, and $x_{ij}$ is the count of the risk alleles (0, 1, or 2) at the *i*th SNP in the *j*th individual. We performed a logistic regression analysis on the PRS that was calculated to determine case–control status [42].

#### 4.4.3. Traditional Machine Learning Algorithms

We evaluated the risk prediction performance of six commonly used machine learning algorithms: support vector machine (SVM), random forest (RF), extreme gradient boosting (XGBoost), logistic regression (LR), light gradient boosting model (LGBM), and adaptive boosting (ADA). This evaluation was performed by using the Python Scikit-learn package [43].

#### 4.4.4. Artificial Neural Network

To classify NSCL/P cases, we constructed four ANN models for each given set of SNPs by using the Keras package of TensorFlow [44]. Our ANN contains two dense layers followed by a rectified linear unit (ReLU). We set the number of neurons in each layer to 8, 16, 32, and 64 for the 3-, 10-, 16-, and 92-SNP models, respectively. In addition, we constructed a dense output layer with sigmoid activations to classify NSCL/P and utilized the Adam method for optimization with an initial learning rate of $5\times 10^{-3}$ and a decay rate of $10^{-5}$ [45]. We trained each ANN model in the 100 epochs setting and measured the binary cross-entropy loss to evaluate the model performance. At the end of the training, each set was replaced with the best weight with low validation loss and high training accuracy.

#### 4.4.5. Genetic-Algorithm-Optimized Neural Networks Ensemble

**Figure 3:**
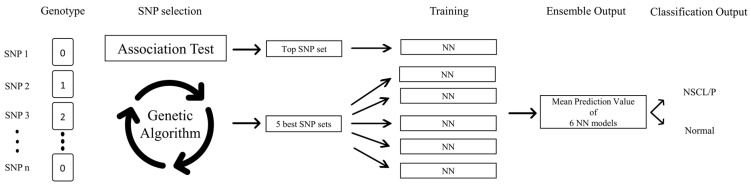
Overview of the GANNE pipeline for NSCL/P classification. Genotype information for each subject is encoded into a n × 1 input matrix, where n is the total number of SNPs. NN, neural network; SNP, single nucleotide polymorphism; NSCL/P, nonsyndromic cleft lip with or without cleft palate.

Our model implemented the GA that was proposed by Tong and Schierz [25] to extract an optimal set of SNPs for classification, followed by an ensemble of ANN results trained with each optimal set. Total cycles and population size were set to 30, and each population consisted of a fixed number of SNPs. To speed up the identification of the local minima, we initialized one population with a set consisting of the most significant SNPs that were found in the association analysis. The goodness-of-fit of the GA was calculated by adding the training loss and the validation loss. For each of the three settings (three, ten, and sixteen SNPs), we created six sets of SNPs, which consisted of five sets from GA and one set from the association analysis. The six SNP sets were trained on each ANN with the same settings as described above. The final value of the ensemble prediction was the average of the prediction values of multiple neural networks (Figure 3).

### 4.5. Model Evaluation and Validation

As evaluation metrics, we calculated the accuracy, which represents the percentage of correctly classified samples, and the area under the receiver operating characteristic curve (AUC). The performance of the ML and DL models was further evaluated using the weighted average F1-score, which balances precision and recall. To address the potential for variability in the results of the ANN models when trained on a GPU server, we repeated the training process 100 times and calculated the average and 95% confidence intervals (95% CIs) of both accuracy and AUC for each model to ensure the reproducibility of the results.

### 4.6. In Silico Functional Analysis

We used the Database for Annotation, Visualization, and Integrated Discovery (DAVID) v6.8 to analyze gene ontology (GO) terms to identify the central function of the SNP markers [46]. We further examined the functional relevance between candidate genes with the protein–protein interaction (PPI) network by using STRING v11 [47].

## 5. Conclusions

GANNE, a deep-learning-based approach for disease risk classification, has shown promise in overcoming the sample size limitations of population-based genetic association studies by utilizing genetic algorithms to select the optimal set of SNP markers. Nevertheless, due to the limited sample size in this study, it is necessary to validate the results in larger, independent Korean populations, as well as to conduct comparative analyses of the model performance across different ethnic groups. With further validation studies, this GANNE model will realize its potential in enhancing NSCL/P genetic risk predictions.

## Figures and Tables

**Table 1:** SNPs associated with NSCL/P in Fisher’s exact test (*p* < 0.05).

Gene	Chr.	SNP	NR/R	RAF (%)	HWE (*p*)	NN/NR/RR		OR	95% CI	*p*-Value
				Case/Control		Case	Control			
*IRF6*	1q32.3-q41	rs2235373 ^a^	A/G	66.8/51.3	0.58	19/57/67	30/56/33	1.91	1.34–2.72	3.5 × 10^−4 c^
		rs2235371 ^a^	T/C	72.4/57.6	0.85	15/49/79	22/57/40	1.93	1.34–2.78	4.4 × 10^−4 c^
		rs2013162 ^b^	A/C	52.8/38.7	0. 57	35/65/43	43/60/16	1.78	1.25–2.52	1.5 × 10^−3^
		rs2235375 ^b^	C/G	52.8/39.1	0.70	35/65/43	43/59/17	1.74	1.23–2.47	2.1 × 10^−3^
		rs1044516	A/C	55.6/42.0	0. 46	31/65/47	42/54/23	1.73	1.22–2.45	2.1 × 10^−3^
		rs595918	G/A	21.5/13.4	1.00	87/49/6	89/28/2	1.76	1.10–2.81	0.02
*RUNX2*	6p21	rs16873348	T/C	35.0/25.6	0.81	59/68/16	65/47/7	1.56	1.07–2.28	0.02
*ARNT*	1q21	rs11204737	C/T	50.7/39.9	0. 45	39/63/41	45/53/21	1.55	1.09–2.19	0.01
*TGFB3*	14q24	rs3917192	G/A	49.0/39.5	0.85	35/76/32	44/56/19	1.47	1.04–2.08	0.03
		rs2284791	G/C	45.1/35.7	0.69	44/69/30	48/57/14	1.48	1.04–2.11	0.03
*MTHFR*	1p36.3	rs3753582	G/T	93.7/88.2	0.20	2/14/127	3/22/94	1.99	1.07–3.69	0.03
*TCOF1*	5q32-q33.1	rs7715100	A/G	9.1/4.2	1.00	119/22/2	109/10/0	2.28	1.08–4.83	0.04

Chr., chromosome; CI, confidence interval; HWE (*p*), Hardy–Weinberg Equilibrium (*p*-value); NR/R, non-risk/risk allele; NN/NR/RR, number of non-risk homozygotes/risk heterozygotes/risk homozygotes according to case–control status; NSCL/P, nonsyndromic cleft lip with or without palate; OR, odd ratio; RAF, risk allele frequency; SNP, single nucleotide polymorphism. SNPs in linkage disequilibrium: ^a^ rs2235373–rs2235371 (*r*^2^ = 0.80), ^b^ rs2235375–rs2013162 (*r*^2^ = 0.98), ^c^ *p*-value less than the Bonferroni-corrected threshold of 0.05 (*p* < 5.4 × 10^−4^).

**Table 2:** Comparison of model performance by using nine risk prediction methods and four sets of SNPs.

SNP (*p*-Value)	Model	Train_Acc	Test_Acc	F1 Score	Test_AUC	Model	Train_Acc	Test_Acc	F1 Score	Test_AUC
3 SNPs (<0.01)	PRS	-	0.584	-	0.625	ANN	0.580	0.597	0.615	0.690
	RF	0.585	0.500	0.500	0.502	*I* ^a^	0.588	0.537	-	0.686
	SVM	0.585	0.597	0.570	0.554	95% CI	0.570–0.590	0.355–0.629	-	0.625–0.724
	XGBoost	0.585	0.581	0.581	0.574	GANNE ^b^	0.725	0.694	0.709	0.741
	LR	0.580	0.597	0.573	0.567	*I* ^a^	0.707	0.597	-	0.720
	LGBM	0.585	0.581	0.581	0.574	95% CI	0.650–0.745	0.500–0.677	-	0.677–0.754
	ADA	0.585	0.500	0.502	0.502	-	-	-	-	-
10 SNPs (<0.05)	PRS	-	0.607	-	0.657	ANN	0.945	0.629	0.649	0.714
	RF	0.955	0.597	0.598	0.597	*I* ^a^	0.880	0.580	-	0.626
	SVM	0.955	0.677	0.678	0.685	95% CI	0.775–0.910	0.484–0.710	-	0.495–0.742
	XGBoost	0.950	0.613	0.613	0.623	GANNE ^b^	1.000	0.742	0.756	0.882
	LR	0.625	0.613	0.614	0.619	*I* ^a^	0.921	0.654	-	0.752
	LGBM	0.935	0.565	0.566	0.568	95% CI	0.870–0.955	0.500–0.823		0.667–0.895
	ADA	0.630	0.630	0.630	0.638	-	-	-	-	-
16 SNPs (<0.1)	PRS	-	0.603	-	0.679	ANN	0.990	0.645	0.659	0.650
	RF	0.995	0.645	0.640	0.635	*I* ^a^	0.935	0.540	-	0.570
	SVM	0.950	0.677	0.678	0.68	95% CI	0.910–0.955	0.403–0.661	-	0.433–0.719
	XGBoost	0.995	0.629	0.630	0.625	GANNE ^b^	1.000	0.694	0.709	0.744
	LR	0.695	0.565	0.565	0.559	*I* ^a^	0.931	0.652	-	0.675
	LGBM	0.995	0.548	0.550	0.549	95% CI	0.895–0.965	0.548–0.774		0.561–0.759
	ADA	0.690	0.581	0.582	0.582	-	-	-	-	-
92 SNPs (All)	PRS	-	0.676	-	0.711	ANN	0.980	0.613	0.633	0.595
	RF	1.000	0.565	0.566	0.592	*I* ^a^	0.865	0.449	-	0.454
	SVM	0.975	0.613	0.615	0.615	95% CI	0.409–0.900	0.274–0.726	-	0.293–0.610
	XGBoost	1.000	0.581	0.578	0.569	-	-	-	-	-
	LR	0.855	0.516	0.518	0.512	-	-	-	-	-
	LGBM	1.000	0.565	0.563	0.555	-	-	-	-	-
	ADA	0.880	0.548	0.550	0.549	-	-	-	-	-

Acc, accuracy; ADA, adaptive boosting; ANN, artificial neural network; AUC, area under the receiver operating characteristic curve; F1 score, weighted average F1 score; GANNE, genetic-algorithm-optimized neural networks ensemble; LR, logistic regression; LGBM, light gradient boosting model; PRS, polygenic risk score; RF, random forest; SNP, single nucleotide polymorphism; SVM; support vector machine; XGBoost, extreme gradient boosting. ^a^ *I* (95% CI) represents the mean and 95% confidence interval obtained from 100 iterations. ^b^ The best model obtained from the six SNP sets selected by GANNE.

## Data Availability

The data supporting the findings of this study are presented in the main text and supplementary material file and additional data can be obtained upon request from the corresponding author. The codes for reproducing the GANNE model used in this study are publicly accessible in the GitHub repository at https://github.com/Osiris2019/GANNE.

## Supplementary Materials

The supporting information can be downloaded at: https://www.mdpi.com/article/10.3390/ijms24054557/s1.
