# Supplementary material for: Genetic Risk Assessment of Nonsyndromic Cleft Lip with or without Cleft Palate by Linking Genetic Networks and Deep Learning Models
Source: Int J Mol Sci. 2023 Feb 25;24(5):4557. doi: 10.3390/ijms24054557 (PMC10003462; doi:10.3390/ijms24054557)
Supplement: Supplementary file 1 [file ijms-24-04557-s001.zip › ijms-2214187-supplementary.pdf]

International Journal of
Molecular Sciences

MDPI

Table S1. Pairwise linkage disequilibrium estimated among 92 candidate SNPs ($r^2 > 0.8$)

Chr.	SNP - SNP	r^2	Chr.	SNP - SNP	r^2
1	rs1537514 - rs2274976	1.00	2	rs765871 - rs3771498	0.89
	rs2235375 - rs2013162	0.98	4	rs698 - rs1693482	1.00
	rs3737967 - rs1537514	0.96	5	rs7713638 - rs7715100	1.00
	rs3737967 - rs2274976	0.96	6	rs910586 - rs2819861	1.00
	rs1537514 - rs3753582	0.93	11	rs3935406 - rs10892434	0.87
	rs2274976 - rs3753582	0.93	14	rs3917192 - rs3917187	0.81
	rs3737967 - rs3753582	0.89			
	rs2235373 - rs2235371	0.80			

Chr., chromosome; r^2, r-squared measure of linkage disequilibrium; SNP, single nucleotide polymorphism

Table S2. Number of SNPs used in the models for NSCL/P risk prediction in three SNP settings using genetic algorithm neural-optimized networks ensemble method.

Gene	SNP ID	NR/R	RAF (%)	OR	*p*-value	GANNET (Count)		
			Case/Control			16-SNPs	10-SNPs	3-SNPs
MTHFR	rs1537514	G/C	0.94/0.89	1.83	0.06	3	1	-
	rs1801133	C/T	0.43/0.40	1.13	0.53	1	4	-
	rs3753582	G/T	0.94/0.88	1.99	0.03	1	2	-
	rs9651118	T/C	0.35/0.32	1.12	0.58	-	4	-
	rs1801131	C/A	0.85/0.79	1.50	0.09	2	-	-
	rs2274976	A/G	0.94/0.89	1.83	0.06	1	-	-
ABCA4	rs481931	A/C	0.64/0.57	1.32	0.13	4	2	-
	rs4147871	T/C	0.05/0.03	1.37	0.51	1	-	-
ARNT	rs11204737	C/T	0.51/0.40	1.55	0.01	2	1	1
	rs16827741	C/T	0.09/0.08	1.27	0.53	3	-	1
	rs7517566	A/G	0.81/0.75	1.45	0.09	1	-	-
IRF6	rs1044516	A/C	0.56/0.42	1.73	$2.1x10^{-3}$	2	4	3
	rs17317411	T/C	0.03/0.03	1.04	1.00	2	1	-
	rs2013162	A/C	0.53/0.39	1.78	$1.5x10^{-3}$	4	6	1
	rs2235371	T/C	0.72/0.58	1.93	$4.4x10^{-4}$	2	1	1
	rs2235373	A/G	0.67/0.51	1.91	$3.5x10^{-4}$	1	2	1
	rs595918	G/A	0.21/0.13	1.76	0.02	1	3	-
	rs2235375	C/G	0.53/0.39	1.74	$2.1x10^{-3}$	1	-	1
	rs599021	A/C	0.31/0.26	1.29	0.21	2	-	-
TGFA	rs3755377	C/T	0.47/0.40	1.29	0.16	-	1	-
	rs3771485	G/C	0.43/0.38	1.26	0.21	4	2	-
	rs3771498	A/G	0.41/0.34	1.35	0.10	1	-	-
	rs3821272	C/T	0.72/0.66	1.30	0.18	2	-	-
	rs11466212	C/T	0.91/0.89	1.23	0.56	-	-	1
MSX1	rs3821949	G/A	0.48/0.47	1.02	0.93	1	-	-
	rs4464513	T/G	0.72/0.71	1.05	0.85	4	-	-
ADH1C	rs2241894	G/A	0.16/0.14	1.22	0.46	1	-	-
TCOF1	rs7715100	A/G	0.09/0.04	2.28	0.04	1	1	1
	rs15251	T/C	0.84/0.80	1.32	0.25	2	-	-
	rs2255796	C/T	0.44/0.42	1.07	0.72	-	-	1
RUNX2	rs16873348	T/C	0.35/0.26	1.56	0.02	4	4	1
	rs1934328	T/A	0.77/0.70	1.45	0.07	5	-	-
	rs2819861	T/C	0.96/0.92	1.98	0.09	3	-	-
VAX1	rs7078160	G/A	0.52/0.50	1.09	0.66	-	2	-
PVRL1	rs7103685	C/T	0.66/0.63	1.15	0.46	-	4	1
	rs906830	T/C	0.55/0.50	1.18	0.38	1	2	-
	rs7129848	C/T	0.24/0.21	1.25	0.3	3	-	-
	rs7940667	A/C	0.97/0.95	1.78	0.2	2	-	-
	rs931953	A/G	0.47/0.47	1.01	1.00	1	-	-
	rs10790330	G/A	0.50/0.45	1.24	0.22	-	-	1
PAX9	rs2295221	A/G	0.06/0.05	1.03	1.00	1	1	-
	rs11156925	G/A	0.70/0.70	1.03	0.92	2	-	-
	rs17104939	C/T	0.26/0.26	1.03	0.92	1	-	-

	rs17104944	T/G	0.78/0.76	1.09	0.68	2	-	-
TGFB3	rs2284791	G/C	0.45/0.36	1.48	0.03	4	2	-
	rs3917192	G/A	0.49/0.40	1.47	0.03	1	5	2
	rs4252328	G/A	0.50/0.43	1.35	0.09	5	1	-
	rs3917211	A/G	0.34/0.30	1.2	0.35	1	-	1
MAFB	rs13041247	C/T	0.59/0.51	1.42	0.05	1	-	-
	rs6029273	A/G	0.41/0.36	1.21	0.32	3	-	-
TBX22	rs11796677	T/C	0.50/0.46	1.16	0.53	3	1	-
	rs1429591	C/A	0.37/0.30	1.41	0.13	1	3	-
	rs195291	T/A	0.89/0.84	1.47	0.23	2	-	-

GANNET, genetic algorithm neural network ensemble technique; NR/R, non-risk/risk allele; OR, odds ratio; RAF, risk allele frequency; SNP, single nucleotide polymorphism. *P*-values were obtained by the Fisher's exact test using PLINK.
